# Robust imaging using electrical impedance tomography: review of current tools

**DOI:** 10.1098/rspa.2021.0713

**Published:** 2022-02

**Authors:** Benoit Brazey, Yassine Haddab, Nabil Zemiti

**Affiliations:** LIRMM, Univ Montpellier, CNRS, Montpellier, France

**Keywords:** electrical impedance tomography, review, robustness, precision

## Abstract

Electrical impedance tomography (EIT) is a medical imaging technique with many advantages and great potential for development in the coming years. Currently, some limitations of EIT are related to the ill-posed nature of the problem. These limitations are translated on a practical level by a lack of genericity of the developed tools. In this paper, the main robust data acquisition and processing tools for EIT proposed in the scientific literature are presented. Their relevance and potential to improve the robustness of EIT are analysed, in order to conclude on the feasibility of a robust EIT tool capable of providing resistivity or difference of resistivity mapping in a wide range of applications. In particular, it is shown that certain measurement acquisition tools and algorithms, such as faulty electrode detection algorithm or particular electrode designs, can ensure the quality of the acquisition in many circumstances. Many algorithms, aiming at processing acquired data, are also described and allow to overcome certain difficulties such as an error in the knowledge of the position of the boundaries or the poor conditioning of the inverse problem. They have a strong potential to faithfully reconstruct a quality image in the presence of disturbances such as noise or boundary modelling error.

## 1. Introduction

Electrical impedance tomography (EIT) is an innovative imaging tool widely employed among the scientific community over the last decades. EIT requires compact and inexpensive equipment compared with other imaging modalities such as Magnetic Resonance Imaging or Computerized Tomography scan, and is found to be safe for the patient. These advantages made EIT popular for many biomedical applications such as chest [1] and brain [2] examination. However, EIT suffers from a relatively low spatial resolution, and requires a good knowledge of the experimental parameters such as the exact position of the electrodes. This required knowledge about the parameters can be seen as a lack of robustness. Many contributions have been made in the scientific literature in order to improve precision of the reconstruction but also to make it more robust, i.e. ensuring precision in the presence of modelling errors. However, many of the tools providing robustness are still unconventional or even rarely used. Although EIT is constantly advancing, its use is often confined to applications exhibiting certain simplifying assumptions. These assumptions can be a two-dimensional representation, a restriction to a bounded domain or even cases where electrode positions can be known with a good precision and where they can be regularly distributed. Thus, the inverse problem, aiming to reconstruct the admittivity of a body, can be solved despite its ill-posed nature.

These examples emphasize that the goal of many works is to obtain good precision for a particular application case. The conventional EIT is currently not robust to modelling errors. This is particularly true in clinical applications where the experimental parameters are difficult to control. To cite a few examples, in [3], limitations and challenges due to a lack of robustness in stroke volume and pulmonary artery pressure examination are presented. In particular, the issue of electrode belt displacement and electrode detachment are highlighted. In [4], problems due to electrode positions, among others, are also discussed. Several reviews focus on recent advances in EIT algorithms, techniques, etc. [5–7], and can be consulted for additional information. In this review, the objective is to highlight tools developed over the past decades that can improve the robustness of EIT, and thus enable or contribute to the achievement of high-performance imaging even with significant modelling errors or constraints.

This paper presents the most common tools for robust EIT. It is organized as follows: first, the usual resolution tools are briefly presented. They aim to provide an understanding of how EIT is commonly performed. Performance criteria are defined and provide a framework for the study. Robust data acquisition and processing methods are then presented in correlation with these criteria. Difference EIT, a popular choice in many applications, is studied, in particular regarding its contributions in terms of robustness as well as its limitations. Tools from the literature that bring precision and robustness to the EIT will be presented, first at the acquisition level and then at the data processing level. The objective is to determine the potential of recently developed tools for a robust EIT, i.e. to provide an image in the presence of significant model uncertainties. Finally, the last section concludes on the state of the art for robust EIT and gives perspectives.

## 2. Usual resolution tools in EIT

EIT allows to map the admittivity of a medium. Information on medium properties is obtained from a multitude of impedance measurements made with a large number of electrodes, providing a large amount of data. In this section, some conventional resolution tools are presented. The set of robust tools and methods will be presented in the following sections. The direct problem, for a given admittivity distribution inside a body, consists in determining the corresponding electric potential at the electrodes. In EIT, the aim is to solve the inverse problem, i.e. to provide a map of the admittivity distribution in the body from electrical measurements [8]. Assuming an operational hardware, reconstructing the admittivity of a medium by EIT can be summarized in five steps:

— defining a current injection/voltage measurement strategy,
— acquiring voltages,
— numerically modelling the body with the electrodes,
— defining a regularization method and implementing it,
— defining an inversion method and applying it.

In a common application case including a large number of electrodes, using all possible pairs for current injection and voltage measurement is irrelevant. Some information is indeed redundant. A simple and often relatively efficient method is to apply the so-called adjacent pair drive, consisting in using only adjacent pairs for injection and measurement. Data acquisition requires a hardware, the main elements of which are a signal generator and an acquisition unit, and a commutation stage allowing to address four among the N electrodes of the body boundary (two for current injection and two for voltage measurement). Injection and measurement electrode pairs are sequentially switched, which allows to obtain a large number of measurements. A numerical model is commonly used and discretizes the domain into small elements so that an approximate solution is obtained. A general approach is to perform difference imaging, the aim of which is to find a stable value of resistivity ρ that minimizes the difference between measured voltages U and reference voltages U(ρ):

2.1 $$\underset{\rho }{\min}\{||U-U(\rho )|{|}^{2}\}.$$

Reference voltages can be obtained experimentally at a different time, and only changes in the medium will theoretically appear once the resistivity is reconstructed. Another approach for difference imaging is to use a numerical model that can simulate potential values on the electrodes. This is generally done by assuming the medium homogeneous, the resistivity of which is the assumed resistivity of the largest surface/volume fraction of the medium, and then extracting the induced voltages after application of currents.

Solving the inverse problem consists in calculating the resistivity distribution when the injected current is known and the voltages are measured at the electrodes. The inverse problem is ill posed: the solutions are not unique, due to the nature of involved physical phenomena and the limited amount of data. The problem is generally regularized to obtain an acceptable solution. Regularization consists in adding information to a problem, which usually reduces the complexity of the model. Regularization is done thanks to the introduction of *a priori* information on the solution. A largely employed regularization tool in EIT, and more generally for the resolution of ill-posed/inverse problems, is the regularization of Tikhonov [9]. In order to favour a particular solution endowed with properties which seem relevant, the regularization term is introduced in the minimization:

2.2 $$\underset{\rho }{\min}\{||U-U(\rho )|{|}^{2}+\alpha ||L\rho |{|}^{2}\},$$

where *α* is here the regularization hyperparameter and L a regularization matrix. This method allows to improve the conditioning of the inverse problem. The employed resolution method in EIT mainly depends on the desired performance in terms of speed and accuracy. It also depends on the type of regularization used. Numerous inversion methods are proposed in the literature, these will be described later. The resolution is most often carried out iteratively: after resolution, the discretized model is updated. When the solution provides voltage values at the level of the electrodes sufficiently close to the values obtained experimentally, the associated resistivity distribution is retained. An open-source software, EIDORS [10,11], contains a wide range of tools for solving EIT problems.

## 3. Tools for robust imaging

### (a) Study framework

This review aims at describing works that may potentially contribute to improve static reconstruction performances. Static reconstruction indeed theoretically allows to obtain the best results in terms of error. Dynamic imagery as well as associated constraints will not be treated.

### (b) Performance criteria

Generally speaking, *robustness* of a system can be defined as its ability to maintain good performance in the presence of external conditions (called disturbances) of high amplitude. These disturbances represent a difference between the theoretical and the real model. For an EIT system, from challenges commonly cited in the literature [3,4,12], robustness criteria can be of the following forms:

— *Robustness against error on spatial localization of borders and electrodes*
Modelling boundaries and electrode position on these boundaries is often challenging in practice, in particular in the medical field where each patient has a specific morphology. Popular methods like difference imaging allowing to overcome this issue are not always applicable, in particular in the absence of time variation inside the domain. Developed tools allowing to consider uncertainities about boundaries will thus be investigated.
— *Robustness against non-optimal electrode configuration*
Unbounded domains, often associated with congestion that forbids some areas of the boundaries to integrate electrodes, are unconventional in EIT due to the difficulty to reconstruct admittivity in the areas where electrodes are few or absent. Theoretical tools and performed experiments will be presented to enhance expectable future improvements.
— *Robustness against noise and bias*
Measurement noise, contact impedance, faulty skin/electrode contact, etc: many sources of external disturbances can affect the reconstruction. Tools allowing to reject or overcome these disturbances will be investigated at both hardware and software levels.

**Figure 1.**
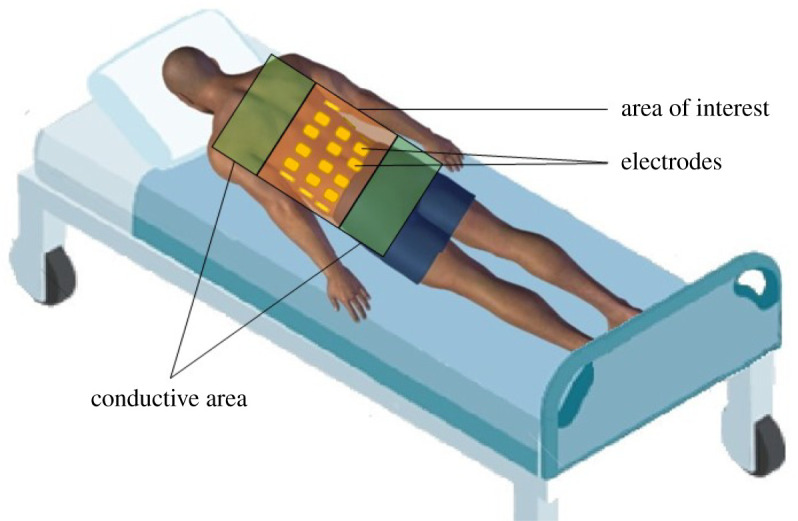
Illustration highlighting some reported issues induced by a lack of robustness in EIT. In this case, the electrodes are not placed in a precise manner, are partially distributed (absence at the level of the belly), and domain modelling must be truncated. (Online version in colour.)

A robust reconstruction by EIT is therefore a successful reconstruction in these different circumstances. An illustration of some common issues that point out a lack of robustness in EIT is given in figure 1. In this hypothetical case, the irregular placement of the electrodes and congestion in particular will lead to modelling errors as well as a lack of sensitivity in some regions.

As mentioned earlier, robustness describes the ability of the system to maintain good performance despite disturbances. These performances can be assessed in different ways, the most relevant probably being precision. *Precision* of an EIT system is regularly employed to represent a global performance of a system. It is expressed in per cent and corresponds to the normalized difference between the reconstructed and real admittivities:

3.1 $$p=\frac{\gamma -\Delta \gamma }{\gamma }.100,$$

with *γ* the admittivity vector and Δγ the admittivity difference vector. Precision depends on many factors at both hardware and software levels.

Robustness can be improved by selecting appropriate tools for data acquisition and processing. The following sections aim at describing such tools from recent literature.

### (c) Difference EIT

#### (i) Description

**Figure 2.**
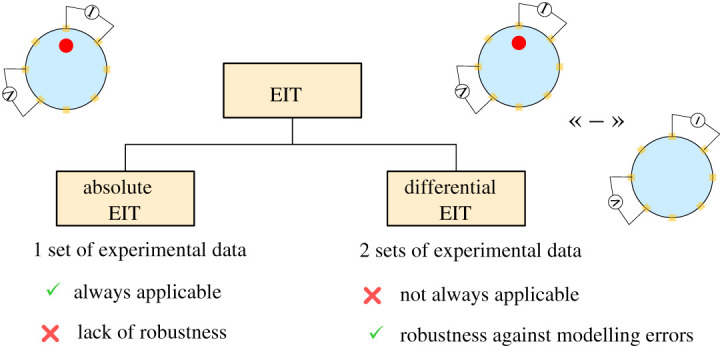
The two subcategories of EIT. Absolute EIT can always be used but lacks robustness. Differential EIT is more robust but requires particular conditions to be applied. These conditions are the possibility of making measurements with and without the object to be detected (in time differential imaging), or distinct frequency electrical properties (in frequency differential imaging). (Online version in colour.)

**Figure 3.**
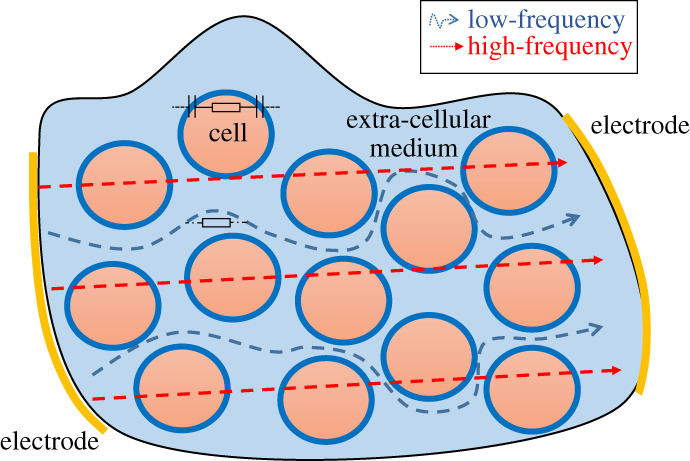
Illustration of the path of alternative electrical current at low and high frequencies through biological tissues. (Online version in colour.)

**Figure 4.**
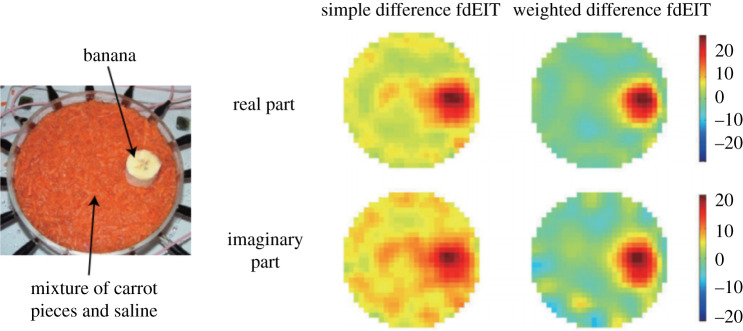
Images reconstruction from a phantom using fdEIT and wfdEIT. The fdEIT method produces larger artefacts compared with the use of the weighted difference method. Source: from [20]. © Institute of Physics and Engineering in Medicine. Reproduced by permission of IOP Publishing. All rights reserved. (Online version in colour.)

EIT can be divided into two distinct subcategories: absolute and differential EIT, as shown in figure 2. Each of these subcategories has its own specificities. Absolute EIT exploits a single set of experimental data in order to reconstruct the body admittivity. Although still theoretically applicable, this technique lacks robustness and is not yet mature. In differential EIT, the difference between two datasets is minimized. This technique is more robust against modelling errors such as an error on the electrode positions. Solving the inverse problem is generally computationally lighter as well. The objective is to calculate the difference between two voltage sets, then to find the admittivity distribution that minimizes this difference. Used data in differential EIT can be obtained in different ways. The first way is to obtain one set experimentally and another one with numerical simulations that serves as reference data. A popular and more robust way is time-difference EIT (tdEIT). Here, the two sets are experimentally obtained at a different time. Although having many advantages, one drawback is that a time evolution is necessary. tdEIT is therefore only applicable to specific application cases. For example, in lung imaging, it is *well suited to trace time-varying physiological phenomena like lung ventilation and perfusion* [1]. Another possibility to perform difference EIT is to acquire reference data using a homogeneous medium beforehand [13,14]. Alternatively to tdEIT, a technique allowing to face modelling errors emerged more recently: frequency-difference EIT (fdEIT). Although this tool is not yet mature, it is very promising, and makes it possible to overcome constraints associated with conventional differential measurement (tdEIT). The principle here is no longer to substract data acquired at different times. The difference in the measured voltages is obtained using different frequency excitation signals. Briefly, at low frequencies (typically less than 100 kHz), the highly capacitive cell membrane prevents electrical currents from penetrating the cell, which will thus mainly pass through the extracellular medium. At higher frequencies, the current passes through both the extracellular medium and the cells, as illustrated in figure 3. The difference between signals measured at low and high frequencies thus depends on the cell sizes and medium and cell electrical properties. More detailed explanations are available in the literature [15]. The electrical response as a function of frequency being specific to each tissue, these differences can thus lead to the reconstruction. Discussions about potential applications of fdEIT are available in the literature [16,17]. fdEIT is therefore suitable, for example, for cancer detection, cancerous cells having different electrical properties than healthy ones [18], with a difference being a function of the type and stage of the cancer. Although fdEIT is quite practical, sensitivity will depend on the difference between medium electrical properties at different frequencies, which may be insufficient for some application cases. In [19–21], an algorithm was developed and later validated with experimental data. The robustness of the approach against boundary modelling errors is highlighted. Also, the authors propose to use weighted frequency differences of complex voltage data with a complex sensitivity matrix to properly handle the interplay of conductivity and permittivity values upon measured complex voltage data. Denoting $u_{\omega }$ the voltages on the electrodes at angular frequency *ω*, the principle of weighted frequency difference EIT consists in calculating the difference:

3.2 $$u_{\omega_{1}}-\alpha u_{\omega_{2}}\;\text{with}\;\omega_{1}\neq \omega_{2},$$

where *α* is a parameter determined from the assumed background admittivities at pulsations $\omega_{1}$ and $\omega_{2}$. The use of this difference with a complex sensitivity matrix allows to minimize undesirable effects of modelling errors. Simulations and experiments exhibit better results with the weighted fdEIT (wfdEIT) than with the simple fdEIT, as highlighted in figure 4. In [22], presented results validate the method in three dimensions. The author demonstrated that the method provides good results in terms of contrast reconstruction for the case of a simple inhomogeneous background. More complex inhomogeneous background cases remain to be investigated to fully validate the method. In a general way, the efficiency of the method depends on the admittivity contrast of the anomaly with respect to the background at different frequencies. wfdEIT thus gives reconstruction that is robust against modelling errors, and the two required datasets can be acquired easily. In recent work [23], a new approach is proposed to improve conventional wfdEIT: the calibrated fdEIT. The principle consists in using an equivalent circuit model of the system (electronics+electrodes+body) to compensate for the measurement errors. The author takes into account a capacitive coupling between the terminals of the voltmeter and the input of the voltmeter which is a virtual ground. This capacitive effect induces, among other things, a dependence of the measurement on the electrode-body contact impedance. The method is particularly useful in miniature EIT imaging where voltage variations introduced by the samples can be lower than measurement errors. Validation was performed in simulation and experimentally, making this technique a promising tool.

#### (ii) Summary and discussion

Difference EIT in its two forms tends to face modelling errors, as summarized in table 1. In particular, fdEIT is very promising, potentially allowing reconstruction without time variation. However, the method is not mature, requires extra hardware and sensitivity of fdEIT, depends on the electrical frequency properties of the tissues being studied, and may not be sufficient in some cases. Making the absolute EIT a robust tool is an important challenge. The next sections present the main tools in the literature that can be associated with it.

### (d) Acquiring voltage measurements

#### (i) Description

The data acquisition method being directly related to the quantity of information collected and used for reconstruction, it is critical to obtain a good precision. Data acquisition is performed from an electronic device that injects electrical signals into a body and then measures the induced voltages. The quality of the reconstruction will depend on the quantity and quality of the acquired data. In particular, the injection and measurement strategies, the quality of the measurements, etc., will all have an impact on the final result.

*Injection and measurement*. If two electrodes seem sufficient to inject a current and measure the corresponding voltage inside the body, this choice is not actually relevant in many cases. The measured impedance will actually be equal to the sum of the impedance of the tissue and the impedances resulting from the contact between the electrodes and the tissue, called contact impedance. This impedance is due to the so-called ‘double layer’ effect, mainly capacitive. The contact impedances being unknown and frequency-dependent, a different technique using four or more electrodes, called multi-pole measurement, is generally preferred. This consists in using distinct electrodes for current injection and voltage measurement. While two-pole measurement is simpler to implement, four-pole measurement, for example, provides better results in most application cases, because contact impedances do not influence the measurements, provided that the input impedance of the measuring device is high compared with them [24].

**Figure 5.**
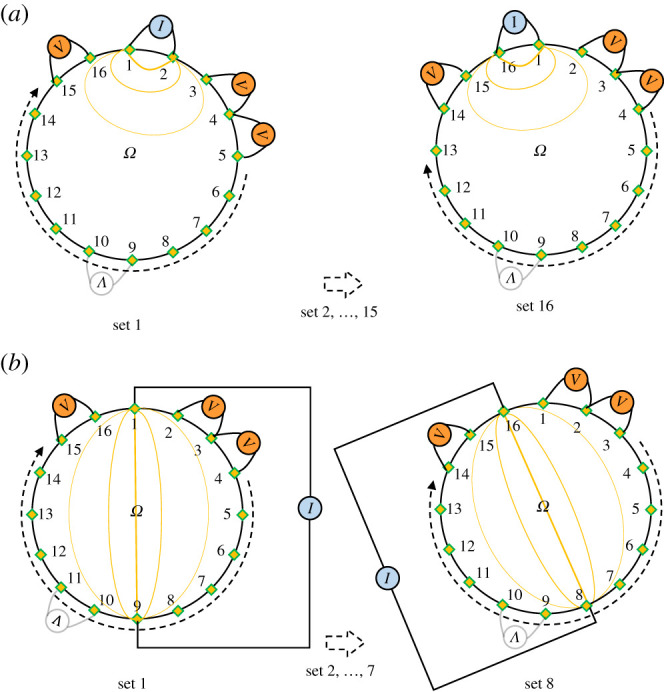
Illustration of classic data collection strategies in EIT: the adjacent pair drive and the opposite pair drive. (*a*) Adjacent pair drive. (*b*) Opposite pair drive. (Online version in colour.)

Different current injection and measurement methods exist in multi-pole measurement. This involves defining certain electrode configurations that will be used for current injection, and other configurations that will be used for measurement. The most popular are called *pair-wise injection* strategies, whereby only two injection electrodes are used. Among these methods, the most commonly used are the *adjacent pair drive* and the *opposite pair drive*. These methods consist in injecting the current with respectively adjacent and opposing electrodes. For both, the resulting voltage is measured for each pair of adjacent electrodes (differential measurement) that can be formed. An illustration of these methods is given in figure 5. These methods are regularly chosen because they are simple to implement and provide relatively effective results: for each area of the medium, there are electrodes having sufficient sensitivity for detection. Adjacent pair drive provides a better sensitivity, but favours detection near the electrodes. Opposite pair drive tends to homogenize sensitivity [25]. In four-pole sensing, the injection method influences the current distribution within the structure and the measurable voltages [26]. It therefore has a direct link with the sensitivity of the system. The spatial resolution is in turn influenced by the measurement method. It is also possible to define injection and measurement strategies based on the angle *α* formed by the electrodes with respect to the centre of a circular area [27]. In order to obtain independent measurements, the following measurement strategy is recommended:

3.3 $$60^{\circ }\leq \alpha =\frac{360}{N}(\mathrm{skip}+1)\leq 150^{\circ },$$

with N the number of electrodes, skip being the number of electrodes included between the two electrodes tested. To the best of our knowledge, optimal current injection and voltage measurement strategies have not been extended to three-dimensional cases. In general, for a planar configuration with regularly distributed electrodes, a pair selection criterion can be defined so as to [27]:

— standardize the distinction of ROIs within the study domain,
— maximize the number of independent measurements, intrinsically linked to spatial resolution reachable by an EIT system [28].

Complex injection and measurement methods can also be implemented in order to optimize the current distribution inside the structure [29]. However, the use of several current sources involves a more delicate phase of equipment calibration. Other injection and measurement possibilities are numerous. For example, it is possible to perform asymmetric measurement, which uses a common reference, instead of differential measurement. However, in order to favour a measurement strategy, the technical constraints should be noted: differential measurements attenuate electromagnetic and electrostatic interference in common mode and have a reduced dynamic range, while asymmetric measurements do not suffer from loss of accuracy due to non-zero common mode amplifier gain. Also, active electrodes, where it is possible to inject current and measure potential in the same electrode, are of interest. They help minimize noise and improve hardware performance while maintaining a small size [30,31]. According to [30], *the key feature of active electrode-based EIT instrument architecture is to have a voltage buffer very closely to the body. This offers several advantages compared with the passive electrode architecture: (1) reduces the sensitivity to electromagnetic perturbation, (2) reduces the need for active and/or passive shielding, (3) stabilizes the input impedance of each electrode and (4) reduces, by using in-electrode multiplexing, the number of cables running from the patient to the main electronics.*

*Managing electrodes*. The number of electrodes to be used in EIT remains an open problem. However, it has been shown that increasing the number of electrodes used tends to improve the resolution of the image obtained, provided that each one is used as a source [26]. By way of example, Zhang *et al.* [13], in their work, highlight the influence of the number of electrodes used to recognize hand movements from differential imaging at the wrist. Increasing the number of electrodes, in this case, increases the accuracy of detection. The gain in precision however decreases when the number of electrodes increases. The number of electrodes used is thus a compromise between precision, measurement time and processing time induced. It should also be noted that increasing the number of electrodes can lead to reduce their size, and thus increase contact impedance. More generally, in order to quantify the theoretical resolution, it is interesting to know that *the best EIT image has a resolution of 32×32 pixels with 32 electrodes* [26]. An order of magnitude of resolution for a two-dimensional circular body surrounded by N regularly distributed electrodes is given by the number of independent measurements: $1/\sqrt{N(N-3)/2}$ [32]. With 16 electrodes, the expected resolution is here about 10% of the diameter. Some elaborated devices were developed, allowing to rotate the electrodes and thus get a large number of measurements with few electrodes [33]. Although this kind of device is interesting for precise reconstruction, it may be difficult to implement it in many application cases. Electrode characteristics are also parameters that significantly influence the quality of the reconstruction. The main challenges related to the electrodes are the insurance of a good contact, the exact knowledge of their position and consideration of the electrode-body contact impedance. Ensuring good contact is particularly delicate for biomedical applications because the electrodes are often placed on a deformable tissue (muscle or fatty tissue). The objective is there to ensure that the electrode conforms to the tissue, does not excessively deform it, and that adhesion is sufficient to maintain a good contact in the presence of disturbances. These disturbances are generally related to movements of the patient, and are sources of modelling errors. Improving the robustness of detection at this level can be done in two ways. The first way is to select electrodes dedicated to the application. The use of flat electrodes is common in the literature. However, in particular for tissue characterization, the electrode-tissue contact may be absent or changing. This induces a variable contact impedance, which can potentially induce large reconstruction errors. An alternative for tissue characterization is to use volume electrodes, for example, spiky electrodes [34]. The advantage of these electrodes lies in the quality of the contact with the tissue. They are however invasive. In order to limit tissue damage, these electrodes should be of reasonable size. Electrode size reduction on the one hand limits the quantity of injectable current, and on the other hand tends to increase the contact impedance, which must be taken into account in order to guarantee the quality of the reconstruction. A second way to ensure measurement robustness is to implement a faulty contact detection [35,36]. In [36], the method allows accounting for faulty electrodes in EIT image reconstruction without *a priori* knowledge of which electrodes are at fault. The method is able to compensate for biased data and to provide high-quality images. It also allows real-time detection of at least one faulty electrode. This method is quite simple to implement and provides good results according to the author. While a punctually biased measurement is not a critical problem, this method alone, without the use of sophisticated electrodes, may be sufficient. At last, in [37], robustness of single-ended measurement to electrode errors is studied. An interesting result indicates that single-ended measurement is more robust and gives better images than differential measurement after compensating for electrode errors in case of faulty contact.

#### (ii) Summary and discussion

The way data are acquired in EIT is not trivial, but yet is essential to obtain relevant information for further admittivity reconstruction. Table 2 summarizes the different evoked tools and their associated characteristics. Robustness of an EIT set-up regarding data acquisition can be characterized by its ability to face modelling errors, and to provide measurements with the necessary amount of information. A work on electrode design and an electrode contact quality detection algorithm allows to ensure signal correct circulation through the circuit and/or to avoid faulty measures. Measurement and injection techniques are well described in the literature, and can be evaluated to provide both spatial resolution and the required information for reconstruction. Some hardwares are proposed in the scientific literature and in trade to allow quality measurements.

**Table 1.** Summary of different proposed tools for precise and robust detection with difference EIT.

method	description	interest in term of robustness and precision	target application
time-difference EIT	two sets of voltages are collected then substracted, the reconstructed admittivity map represents the difference between admittivities acquired at different times	robustness against modelling errors, but not always applicable in practice	time variations of admittivity, boundary modelling errors
frequency-difference EIT	the two sets of data are here acquired with two different excitation signal frequencies	robustness against modelling errors	boundary modelling errors

**Table 2.** Summary of different proposed tools allowing to get data for precise and robust detection.

method	description	interest in term of robustness and precision	target application
multi-pole measurement	Using four or more electrodes simultaneously to perform measurement	four-pole measurement allows reducing measurement errors due to contact. Using more than two injection electrodes increases sensitivity but requires an additional calibration step	All EIT applications
choosing the number of electrodes	trade-off between expected resolution and processing duration, hardware complexity, electrode size …	theoretical resolution directly related with the number of electrodes	All EIT applications
injection strategy	electrodes used to inject current	affects sensitivity	All EIT applications
measurement strategy	electrodes used to measure voltages	affects spatial resolution. A measurement angle (two-dimensional) can be used to get independent measurements	All EIT applications
active electrodes	injection and measurement at the same electrode	reduced noise	All EIT applications
electrode design	spiky, flat, etc.	robustness against biased measurements	All EIT applications
electrode detachment detection algorithm	algorithm allowing to detect if one (or several) electrode/tissue contact is faulty	robustness against biased measurements	All EIT applications

This section aimed at describing different tools allowing to get suitable data. Below, admittivity reconstruction tools are presented.

### (e) Reconstructing the admittivity map

#### (i) Description

Once the data have been acquired, it is necessary to use algorithmic tools in order to reconstruct the admittivity of the body. In [38], an interesting result points out that the resolution of a reconstructed image depends more on the reconstruction strategy than on the drive pattern, which highlights the importance in the choice of the reconstruction method. Some methods are explicitly dedicated to dynamic imaging, such as the maximum a posteriori approach (MAP) [39] Kalman-type filters [40], and are not considered here. The inversion step occurs once datasets are collected. During this step, the admittivity vector *γ* is estimated from voltage measurements U(γ). One of the open challenges of EIT is that in most experiments the body boundaries ∂Ω are not known precisely. Traditional image reconstruction methods assume the boundaries to be known *a priori*, and the only remaining unknowns are the conductivity at each point. The EIT problem is usually solved using an approximate domain $\Omega_{m}$, which is an estimate of the shape of the body Ω. However, it has been noticed that using a slightly incorrect model can lead to serious artefacts and distortions in the reconstructed images, particularly present in absolute imaging. Likewise, technical constraints such as unbounded domains or partially distributed electrodes on the boundaries can complicate the resolution. Two types of reconstruction tools are commonly described in the literature:

— regularization,
— inversion algorithms.

In the following paragraphs, these various issues as well as the associated resolution tools are presented.

*Facing the ill-posed nature of the EIT problem*. The ill-posed nature of the EIT problem is reflected in poor conditioning. A small error in the measured voltages results in large reconstruction errors. The ill-conditioning of the inverse problem therefore represents a lack of robustness to measurement errors.

Regularization is a popular choice in EIT. Regularization is commonly associated with the inversion tool to improve the conditioning of the inverse problem by introducing *a priori* data on the solution. It results in the addition of a term of regularization in the functional minimization process, given equation (2.1), which is for this particular case the L_2_ squared norm. Tikhonov regularization is probably the most used regularization method in EIT, and more generally for solving problems which are not well posed as well as for inverse problems. The principle of this regularization was previously presented as equation (2.2). The solution obtained by a Tikhonov-type method is therefore an approximate solution to the ill-posed problem, stable with respect to the data but dependent on the regularization parameter *α* used. There are several techniques for choosing the regularization parameter, such as the Morozov criterion [41], the cross-validation criterion [42] or the L-curve technique [43]. For low values of the hyperparameter *α*, the sensitivity to changes in admittivity is higher than for large values [39], but the regularizing effect is reduced. There is an anisotropic variant of this regularization, more representative of living tissue [44,45]. An alternative to this method mentioned in the literature is Total Variation regularization (TV) [46]. The TV of a conductivity image is defined as follows:

3.4 $$\mathrm{TV}(\sigma )=\int_{\Omega }|\nabla \sigma |\;d\Omega .$$

This method has an ability to preserve edges in reconstructions, which is due to its use of the penalty term of the L_1_ norm, which is discontinuous and therefore not differentiable at any point. This type of regularization is particularly suitable when seeking to reconstruct discontinuous functions, such as, for example, an inclusion such as a tumour, a bone, etc. TV is thus suitable for many biological imaging applications. Expressed simply, piecewise continuous functions can be a solution, which is not the case for Tikhonov regularization. The ability of the method to give sharper results has been proven *in vivo* [47]. It should be noted that TV is more sensitive to measurement noise, but provides satisfactory results for realistic noise amplitudes. Anisotropic [48] and higher-order term [49] variants of TV have also been developed. However, TV often induces a longer calculation time, due to the associated inversion method (associated inversion is described in the previous paragraph). The chosen regularization must be associated with an inversion method. The most widespread derive from Newton’s inversion method, such as Gauss–Newton (GN, also called modified Newton–Raphson) [50]. It should be noted that the quality of the reconstruction depends on the inversion method but also on prior knowledge about the solution. Thus, these methods are not always suitable because they are incompatible with the used prior knowledge characteristics chosen (i.e. the regularization method), such as TV, for example, because of its non-differentiability. TV is well suited for inclusion reconstruction due to its ability to reconstruct discontinuous functions (in the present case, of admittivity). Thus, compatible inversion algorithms have been developed. In particular, three methods of interest: Split Bregman (SB), Primal and Dual Interior Point Method (PDIPM) and Linearised Alternating Direction Method of Multipliers are studied and compared in the literature [51]. From this comparison, the SB and PDIPM methods seem to be appropriate choices due to their stability and relatively high spatial resolution. To conclude, regularization is thus a tool that increases robustness against modelling errors by decreasing the ill-posedness of the inverse problem. TV is suitable for inclusion reconstruction but requires more computational time, which remains most often reasonable for static reconstruction.

**Figure 6.**
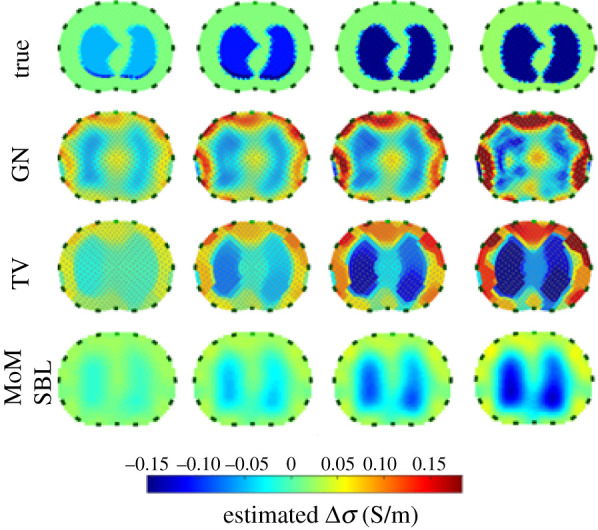
*In vivo* lung EIT reconstructed images using MoM-SBL (Matching of Moment SBL). The SBL method is compared with other algorithms: Gauss–Newton and Total Variation. Source: adapted from [57]. (Online version in colour.)

Some recent inversion methods have also been developed to overcome the ill-posedness of the problem: Sparse Bayesian Learning (SBL). Bayesian learning is based on incorporating *a priori* knowledge of a model. It is useful, for example, when one wants to provide estimates of uncertainty in the model parameters or when there are limited data available for learning a model. SBL was first developed by Tipping [52]. According to Zhang *et al.*, *one attraction of SBL is that, different from the popular minimization based algorithms, whose global minimum is generally not the sparsest solution, the global minima of SBL are always the sparsest one. In addition, SBL have much fewer local minima than some classic algorithms, such as the FOCUSS family* [53]. In structure-aware SBL (SA-SBL), clustered sparsity and intra-cluster correlation allow to obtain better accuracy [54]. The structure-aware modelling capability promotes clustered sparsity and eliminates irrelevant components. It also achieves a higher spatial resolution and improved robustness against Gaussian noise compared with current algorithms. According to the author, the Bayesian probabilistic approach is more suitable than usual regularization techniques to model *a priori* knowledge as it allows quantification of the uncertainity in the recovery. In recent publications, the method is extended for three-dimensional structures [55] and fdEIT, previously mentioned [56]. An illustration of the performance of SBL is given in figure 6. In the presented works, another variant of the SBL, the MoM-SBL (Matching of Moment SBL) is used. This approach undertakes the image reconstruction problem’s nonlinearity, offering robustness to noise and reduced susceptibility in modelling errors. The performance of the method is compared with that of other common methods such as TV. Inclusions are more accurately reconstructed by SBL, with higher correlation. At last, in [58], a learning-based reconstruction algorithm is proposed. It exploits training datasets to generate a low-dimensional manifold of approximate solutions, which allows to convert the ill-posed problem to a well-posed one. The deep learning framework provides a nonlinear regression for the training data, which acts as learning complex prior knowledge of the output. This recent method has an interesting potential due to good results and requires further development.

*Managing uncertainities on body shape and electrode positions*. In the above cited methods, solvers are associated with regularization. In a recent study, regularization-based traditional methods are compared with a D-bar algorithm [59]. An interesting result is that D-bar appears to be much less sensitive to electrode position errors than regularized reconstructions, holding the regularization parameter fixed, in difference EIT. D-bar methods for EIT [60] use nonlinear Fourier transforms specific to the EIT problem. In difference imaging, the method appears to be in particular less sensitive to errors in electrode position than the regularized methods. In a recent publication [61], a D-bar method is paired with a trained convolutional neural network as a post-processing step. The training data are boundary shape independent, which gives significant improvements in image quality. Only two-dimensional simulations being performed, this interesting tool however requires to be developed in further works.

**Figure 7.**
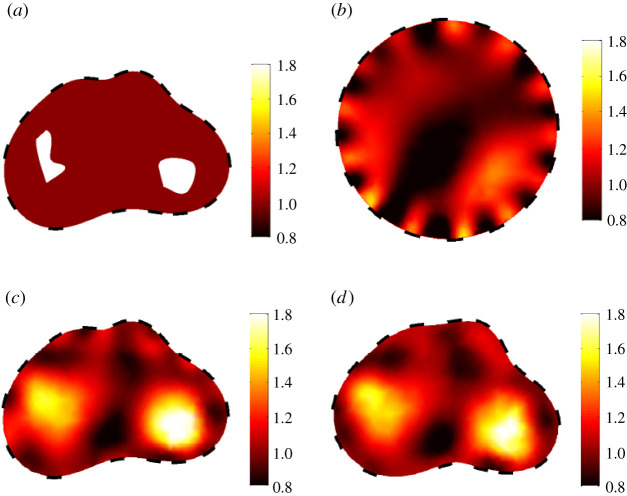
Admittivity and boundaries reconstruction. (*a*) Phantom used in data simulation. (*b*) Reconstruction corresponding to an incorrect fixed geometry. (*c*) Reconstruction corresponding to the exact geometry. (*d*) Simultaneously reconstructed admittivity and measurement geometry. Source: from [70]. Copyright © 2013 Society for Industrial and Applied Mathematics. Reprinted with permission. All rights reserved. (Online version in colour.)

The various works cited below attempt to provide robustness against unknowns concerning domain shape and/or contact impedances by integrating them as uncertainities in the model. In [62], performances of a nonlinear reconstruction approach for tdEIT are studied. It appears that the nonlinear approach produces better estimates of the conductivity change and is robust against modelling errors, at least to the same extent as the conventional linear approach. In pioneering work, a simultaneous conductivity and electrode movement reconstruction has been proposed for differential imaging in [63,64]. These approaches are based on a linearized disturbance model and have only been evaluated for relatively small movements of the boundary between measurement states. [64] is included in EIDORS. The method proposed by Kolehmainen *et al.* [65,66] attempts to compensate for errors caused by an inaccurately known body shape in two-dimensional EIT using the theory of Teichmuller mappings. The method assumes an isotropic conductivity *γ* in the unknown real domain Ω. A model of the domain, $\Omega_{m}$, is used and represents the best possible estimate of Ω. The author shows that it is possible to find a unique conductivity $\hat{\gamma }$ in $\Omega_{m}$ that is as close to isotropy as possible. He finally shows that there is a function of this unique conductivity that represents a distorted image of the original conductivity *γ* in the real domain Ω, and that the distortion depends only on the model error. This method avoids that local errors lead to non-local modifications in the reconstruction. The extension of the method to three-dimensional EIT has been developed in [67]. Later work by the same author takes the anisotropic conductivity model and solves the problem using a Beltrami equation [68,69]. Strong distortions appear, especially near the electrodes for conventional reconstruction, while the image is of good quality with the presented method. In the method proposed by Dardé *et al*. [70–72], the need for prior geometric information is relaxed by introducing a Newton-type least-squares algorithm that simultaneously reconstructs the admittivity distribution and the shape of the object. The method is constructed within the framework of the complete electrode model and is based on the Fréchet derivative of the corresponding current–voltage mapping with respect to the boundaries of the object. The interest of these methods appears clearly on observation of the results for the case where significant errors on the boundaries are present (figure 7). Finally, Nissinen *et al.* [73] apply the Bayesian approximation error approach to compensate for an inaccurately known boundary shape in absolute EIT. In the approximation error approach, the modelling error caused by an inaccurately known boundary is treated as an auxiliary process noise (disturbance) in the measurement model. The probability distribution of the modelling error noise is approximated as Gaussian and estimated using an atlas of body geometries from computerized tomographic images. These statistics are then used in the reconstruction process to compensate for uncertainty in body shape. In [74], the approximation error approach was used to recover an approximation of the domain boundary using the joint distribution of the modelling error and the boundary parametrization. In each of the articles are presented good results for the reconstruction of borders, and appear to work with large modelling errors, which is very promising. They would be worthy to be exploited in further works. It would indeed be technically desirable to show that absolute imaging can provide good results despite modelling errors, because absolute EIT is the simplest to implement experimentally.

**Figure 8.**
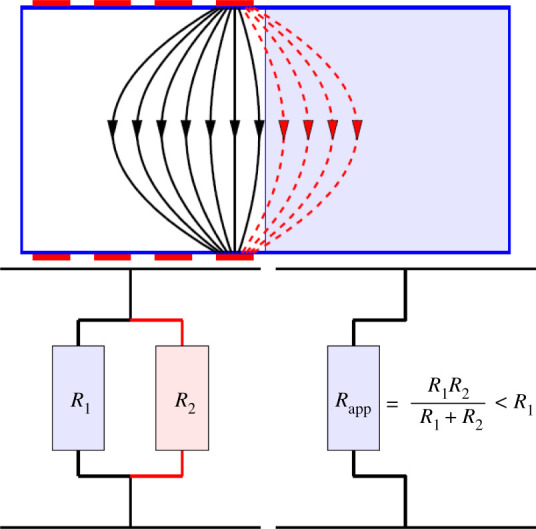
Schematic illustration of the apparent change in conductivity related to the truncation of the domain. Source: from [76]. Reproduced by permission of Inverse Problems and Imaging. (Online version in colour.)

*Unbounded domain*. Another difficulty encountered concerns unbounded domains. In most current EIT medical applications, boundaries are defined by a conductive and non-conductive medium interface, which is the tissue–air interface delimiting the human body from its environment (for example, the contour of the thorax or of the wrist). The following boundary condition is then defined:

3.5 $$J\cdot n=0\;\text{on}\;\partial \Omega \setminus \overset{L}{\underset{l=1}{⋃}}e_{l},$$

with *J* the current density, *n* the outgoing normal unit vector and *e_l_* the electrodes. This means current density following the outgoing normal is assumed to be zero at the boundaries except at the electrodes. The definition of ∂Ω becomes more complex when the studied area is not an isolated surface/volume, i.e. when the current can circulate far beyond the region of interest due to the absence of insulating boundaries. Delimiting the domain is then a non-trivial task. The effect of domain truncation has been studied [75–77], an illustration of which is given in figure 8. When the domain is truncated, the apparent resistance perceived by the measurement system is no longer the real resistance but can be modelled as a resistance mounted in parallel with the real resistance. If the resistance of the modelled area is not negligible compared with the actual resistance, the total resistance is lower, giving the appearance of an area of greater conductivity. This will induce the presence of artefacts on the reconstructed conductivity map. In other words, if the model is truncated too close to the electrodes, errors are produced in the reconstructed images. On the other hand, if the model is extended very far from the electrodes, the calculation time may become too long in practice. The approach proposed in [76], based on a partial Dirichlet-to-Neumann mapping, allows to compute reconstructions in a subdomain with almost the same accuracy as reconstructions that are computed in the full domain at much reduced computational costs.

**Figure 9.**
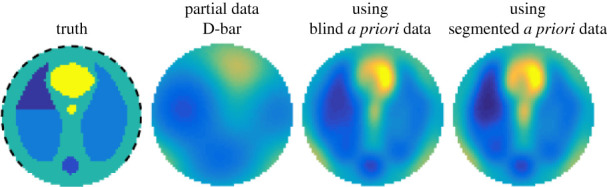
Example simulating a reconstruction with partially distributed electrodes (pneumothorax in the left lung). The simulated noisy measurement is collected from 75% ventral data. The first image displays the true conductivity with the position of electrodes indicated. Using a partial data D-bar approach alone results in a reconstruction with low spatial resolution, where the pathology can be hardly seen (second). Incorporating *a priori* data corresponding to a healthy patient directly in the reconstruction method significantly improves the spatial resolution (third). Refining the *a priori* data improves the reconstruction further, allowing even sharper visualization of the pathology (fourth). Source: from [80]. Reproduced by permission of Inverse Problems and Imaging. (Online version in colour.)

*Partially distributed electrodes*. Hauptmann *et al.* [78,79] have proposed reconstruction tools taking into account a domain where electrodes are partially distributed on the boundaries. This type of tool can be useful when congestion forbids the use of electrodes all around the body, for example, for an examination in intensive care where it is impossible to move the patient and thus to access a part of his body with electrodes. In their works, the reconstruction is based on a truncated and linearized D-bar method. Data are modelled as a partial map from Neumann to Dirichlet (ND map). These data are compared with the ND map including all the boundaries. The error linearly depends on the size of the missing part of the boundary. There is also the same linear dependence regarding the difference in conductivities reconstructed from the partial and full boundary data. The Neumann problem is considered rather than the Dirichlet problem, since the partially supported Dirichlet boundary conditions are not representative of a physical system in many applications. In this context, the author introduced a partial ND map, represented as a composition of the ND map and a partial boundary map. It is shown that the choice of the partial boundary map is crucial for the error analysis and the quality of the reconstruction. In [80], a D-bar method is also exploited to reconstruct the domain conductivity from partial boundary measurements (figure 9). As previously for the reconstruction of boundaries, the implementation of this type of method is computationally heavy but would be a great improvement for partially distributed electrodes application cases where static reconstruction is desired.

#### (ii) Summary and discussion

Reconstructing the admittivity map can be challenging in many non-trivial application cases. The main reported difficulties come from the ill-posed character of the problem, unbounded domains, partially distributed electrodes and modelling errors due to the unknown exact boundary shape. Table 3 summarizes the different evoked tools and their associated characteristics to overcome these issues. The reconstruction algorithm used will have a great impact on the accuracy of the reconstruction. The associated regularization strategy will also have an impact on the precision because this one enables to make assumptions *a priori* on the form of the solution. Yet, the proposed tools are also remarkably robust against the cited difficulties because they allow the reconstruction of truncated or partially inaccessible domains. It can also be noted that the D-Bar method gives promising results in several works making it possible to improve the robustness of EIT against modelling errors. There is therefore a strong potential for improvement in EIT that deserves to be highlighted in the future, notably with tests on biological tissues.

**Table 3.** Summary of different proposed tools allowing to reconstruct the admittivity map for precise and robust detection.

method	description	interest in terms of robustness and precision	target application
SA-SBL	inversion algorithm	reducing the ill-posed character of the inverse problem: robustness against noise and bias	All EIT applications
regularization	adding *a priori* information in order to simplify the problem	reducing the ill-posed character of the inverse problem: robustness against noise and bias	All EIT applications
boundary reconstruction algorithms (Teichmuller mapping, D-bar, etc.)	inversion algorithms	provides a reconstruction that is robust against modelling errors. Seems efficient even with large boundary modelling errors. Particularly useful when difference EIT cannot be performed	large model uncertainities about body shape and electrode positions
partial Neumann-to-Dirichlet mapping	inversion algorithm	robust algorithm against modelling errors when the domain is truncated	unbounded domain
D-Bar method	inversion algorithm	robust algorithm against modelling errors when electrodes are partially distributed. Allows reconstruction when electrodes cannot surround the boundaries with a higher precision	congestion forbids full-boundary electrode position model uncertainties about electrode positions

## 4. General conclusion and discussion

This paper was devoted to the study of the state of the art in EIT that could make this technology more robust, i.e. allowing accurate detection in suboptimal cases that have been little explored so far. Indeed, if EIT allows the reconstruction of quality images in many applications, the reconstruction becomes delicate in non-standard cases, for example, when some experimental parameters cannot be controlled with precision. Thus, in this paper, the objective was to highlight tools that can be used in a systematic way to enable successful image reconstruction in the presence of model uncertainties. Data acquisition and processing tools were thus studied, and summarized in the tables 1, 2 and 3. These tables can be used as a basis for selecting robust tools.

As an illustrative example, concerning the acquisition of an EIT image of our patient in a suboptimal configuration (figure 1), some of the developed tools can be selected from the tables. The state of the art associated with these particular problems can thus be studied. For example, Calvetti *et al*. [76] would allow to take into account the truncation of the domain. Similarly, Hauptmann *et al*. [78] (presented in two dimensions) would allow in three dimensions to improve the quality of the reconstruction due to the absence of electrodes on part of the patient’s contours.

The most powerful resolution tools generally induce an increased computational load compared with classical tools. However, this load could be acceptable in the case of static EIT, for example if the objective is to provide a single image by EIT as an alternative to conventional imaging modalities. This could be considered in order to limit the cost of the acquisition equipment, or when the space requirement does not allow their use, as in a clinical operation room. Image registration could thus be performed from preoperative images.

Improving the robustness of EIT against these errors can also be done in several ways. Differential measurement has proven to be a robust tool against moderate magnitude errors on body geometry, contact impedances, electrode position, etc. The advantage of differential EIT is that its robustness is present without a complex algorithm incorporating uncertainties in these parameters. Its most common form, tdEIT, is however not always applicable due to the absence of time variation. The tools described in the literature tend to indicate that other acquisition methods could become good candidates. For example, fdEIT data can be easily acquired because the same body is used for the acquisition of both datasets. Although fdEIT suffers from a generally lower sensitivity and a more delicate calibration procedure, both weighted and calibrated versions of the method provide interesting results.

Interesting robust tools were presented for data acquisition and processing. At the level of data acquisition, defining an injection and measurement strategy according to the application is a mastered method, at least in two dimensions. This affects the sensitivity, and thus the SNR, and also allows for independent measurements. A faulty contact detection algorithm could be used to detect a potential electrode detachment. Probably the most important advances in robust EIT imaging are in data processing. Recent developments allow to consider the use of a method whose application has long been problematic: absolute imaging. They make it possible to integrate an uncertainty on the position of the electrodes, a truncated domain or to reconstruct the admittance when the electrodes are partially distributed around the body. Absolute imaging, associated with these different tools, offers methods with high robustness for the reconstruction. Regularization-based algorithms, such as Newton-based methods, have been shown to improve reconstruction quality. They provide robustness by facing the ill-posed nature of the inverse problem. Some recent inversion algorithms such as SA-SBL were also developed for this purpose, and offer interesting perspectives. Other algorithmic tools allow to manage uncertainities on body shape and electrode positions on the borders. Some algorithms have been developed to deal with suboptimal configurations such as partially distributed electrodes or a truncated domain.

To conclude, robustness against moderate modelling errors is widespread with the use of difference EIT. However, this method is limited to specific application cases. More robust detection tools have been developed, in particular at the algorithmic level with inversion methods such as the D-Bar method or the SA-SBL. They are applicable to absolute EIT and therefore have the potential to make EIT be used in a wider range of applications. The presented techniques and algorithms are very encouraging for robust EIT. In particular, fdEIT and absolute EIT have the potential to perform robust imaging without the need for time variation, but are not yet fully mature. Their development in the coming years is likely to reveal the potential of EIT as a robust tool, which, combined with its other advantages in terms of cost and size in particular, could make it a prevalent imaging modality.

## Data accessibility

This article has no additional data.
